# Finite element analysis confirms the optimal apex position in medial opening wedge high tibial osteotomy to avoid lateral hinge fracture

**DOI:** 10.1002/jeo2.70042

**Published:** 2024-10-16

**Authors:** Humza T. Osmani, Radhika Gupta, Rosemary Earl, Stanisław Tomaszczyk, Tom Turmezei, Neil A. Segal, Michael Sutcliffe, Joel Melton

**Affiliations:** ^1^ Department of Trauma and Orthopaedics Addenbrooke's Hospital Cambridge UK; ^2^ Department of Engineering University of Cambridge Cambridge UK; ^3^ Department of Radiology Norfolk and Norwich University Hospital Norwich UK; ^4^ Department of Rehabilitation University of Kansas Medical Center Kansas City Kansas USA

**Keywords:** apex, fracture, medial, osteotomy, tibia

## Abstract

**Purpose:**

Lateral hinge fracture is a significant complication of medial opening wedge high tibial osteotomy. While fracture risk is closely associated with the osteotomy apex position, the optimum position remains variable within the literature. Our hypothesis is that stresses at the osteotomy apex predicted by finite element analysis can be used to identify an apex position which minimises intra and postoperative fracture risks.

**Methods:**

A finite element model was studied to investigate the effect of varying the hinge position on fracture risk and severity for a given bone geometry; variables analysed included stress, strain and micromotion levels. Nine further knee models were studied to assess the variability between patients' bone properties and examine the effect of apex location on strains.

**Results:**

Lateral hinge width and height significantly influence intra‐operative stress, strain, and fracture risk, while hinge width predominately determines postoperative stability. Wider hinges improve postoperative stability, but increase the likelihood of intra‐articular fractures. Aiming the apex at the fibular head height minimises strain. The osteotomy apex should be located such that the hinge width is equal to 13% of the medial‐lateral width to minimise apex stress and fracture risk while preserving sufficient bone at the hinge for stability. The height of the apex from the tibial plateau should maintain a minimum value of 16% of the medial‐lateral width to avoid intra‐articular fracture, with the apex below the fibula head if necessary. The size of the tibia does not alter the optimal location, making our findings applicable across all tibia sizes.

**Conclusions:**

Our study has investigated and verified a proposed optimal apex position, based upon fracture risk prediction and micromotion at the osteotomy apex. This is clinically useful due to the potential use of the apex point on preoperative 2D radiographs when planning surgery.

**Level of Evidence:**

Not applicable.

**Abbreviations**
2D: two dimensional
3D: three dimensional
CT: computed tomography
FEA: finite element analysis
h: height (tibial plateau to apex)
MLW: medial‐lateral tibial width
MOWHTO: medial opening wedge high tibial osteotomy
OA: osteoarthritis
OAP: optimal apex position
w: hinge width
WBCT: weight bearing computed tomography

## INTRODUCTION

The global age‐standardised prevalence rate of osteoarthritis (OA) of the knee has been estimated as 4380 per 100,000 in 2019, an increase of 122% from the 1990 prevalence rate [16]. A costly but effective treatment is a total knee replacement. However, in younger patients with medial compartment OA and varus malalignment, a medial opening wedge high tibial osteotomy (MOWHTO) is a viable option [6, 25], to realign the load bearing axis of the knee, while preserving bone stock and providing good survival outcomes [9, 21]. Substantial correction angles are needed and it may be beneficial to over or under‐correct depending on the patient‐specific geometry, additionally accounting for correction loss [25]. Neutral alignment has also been reported in the literature to improve Lysholm scores, with no correlation being found between age and scores [19]. A commonly utilised correction angle is ‘1 degree equals 1 mm of opening’, although this has been shown to be inaccurate compared to other planning techniques, such as Miniaci [4].

A key risk for the MOWHTO procedure is lateral hinge fracture [1]. Other risks include delayed unions, nonunions [8, 10] and increased posterior slope [30]. A critical component of a successful MOWHTO is the maintenance of the lateral hinge, which acts as a fulcrum during the osteotomy. The osteotomy apex is generally directed toward the proximal tibiofibular joint on the lateral cortex, where a fracture can occur while making adjustments in the coronal and sagittal planes.

A commonly accepted fracture classification for MOWHTO surgeries was proposed by Takeuchi et al. [28]. It has since been validated [18] as a suitable tool for assigning rehabilitation strategies and an indication of possible further complications. This classification distinguishes between three types of fractures: Type I, type II and type III. Type I fracture extends horizontally from the tibia surface, following the cut plane, type II extends down from the apex towards the bottom of the tibiofibular joint and type III extends up from the apex to the tibia plateau giving an intra‐articular fracture. Type I is the most common (73%), while types II (19%) and III (8%) are clinically more significant [20].

The optimal apex position (OAP) is not well defined. The location should minimise the risk of lateral hinge fractures intraoperatively while ensuring sufficient micromotion during postoperative loading to give effective healing. Currently, there is variation in the recommended values for the lateral hinge width, as well as the distance from the joint line to the apex quoted by surgeons and researchers [15, 17]. Clinical studies can be limited by the challenges of collecting a large clinical sample size for this procedure. Hence we hypothesised that bioengineering models using finite element analysis (FEA) could be used to predict the stresses at the osteotomy apex and hence develop guidelines for the location of the osteotomy apex so as to avoid the risk of clinically significant type III fractures during MOWHTO surgery while ensuring sufficient micromotion for postoperative healing.

## MATERIALS AND METHODS

### Proof of concept

Laboratory experiments were used in the proof‐of‐concept study. The aim of this study was to determine the mechanisms of fracture and corresponding fracture types to allow for the interpretation of the stress results in the FEA models.

The study used artificial bone models (foam cortical shells by Sawbones, Sweden) and two‐dimensional models made from 12 mm thick Perspex. The geometry of the two‐dimensional (2D) Perspex models was chosen to represent a typical coronal plane outline as seen on weight bearing computed tomography (WBCT) scans. Only a proximal part of the tibia was modelled, with sufficient material included to ensure that the stresses at the apex were not significantly affected by the omitted distal part of the bone.

For both models, the osteotomy cut was initiated 40 mm from the joint surface. The apex was positioned 16 mm from the joint line in the artificial bone models and at distances of 13, 16 and 19 mm from the joint line for the Perspex models. For both models, eight hinge widths evenly spaced between 4 and 25 mm were tested.

**Figure 1:**
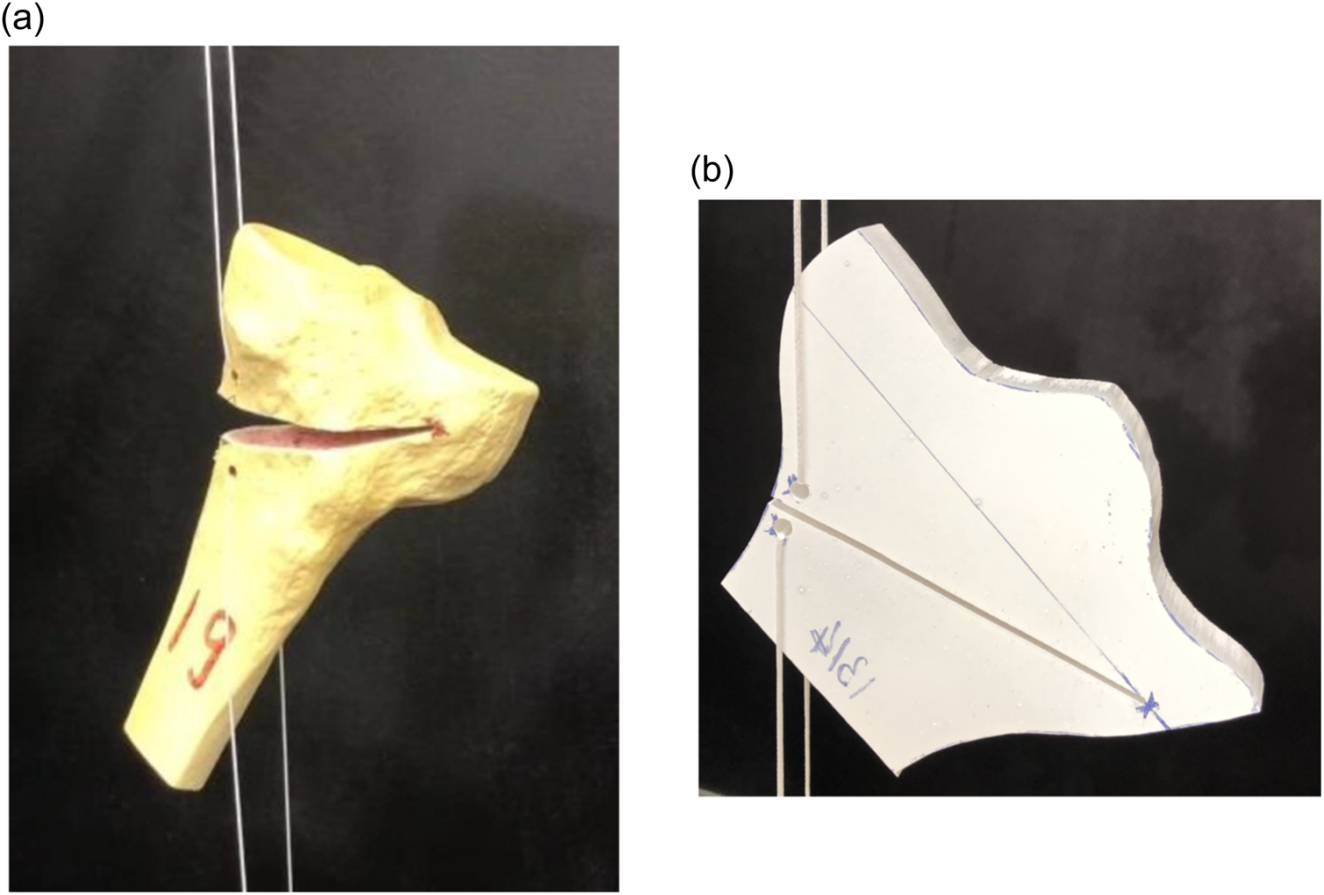
Illustration of the experimental test specimens mounted in the Instron machine. (a) Sawbones 3D model, (b) Perspex 2D model. The maximum medial‐lateral widths were 86 mm and 80 mm for the Sawbones and Perspex specimens, respectively. The image of the Perspex model has been flipped to maintain consistency of osteotomy orientations through the paper.

The load used to open the wedge was applied using an Instron universal testing machine (High Wycombe) via a cord attached to either side of the wedge opening. Figure 1 shows examples of the two types of models mounted in the Instron machine. The load was applied until the tibia fractured, which invariably initiated from the apex. The failure load, the Takeuchi fracture type and the wedge opening angle (defined between the top and bottom edges of the cut in the coronal plane) were recorded for each test.

### FEA modelling overview

The procedure and software used for creating the FE models were as follows: (1) Stradview was used to convert computed tomography (CT) images into three‐dimensional (3D) meshed models; (2) Fusion 360 was used to create the required model geometry, including the osteotomy and mesh; (3) the models were imported into the FEA software Abaqus (Dassault Systèmes), where the element details, material properties, and constraints were assigned and loads were applied. Separate FEA models were constructed for each apex location, using second‐order tetrahedral elements. Further specific details of the models used for our FEA are given in the following sections.

Deformation and fracture of the bone is determined by the local stresses and strains. To characterise the local stress, we used the von Mises effective stress.

### FEA: Part 1

The purpose of this first part of the FEA study was to establish procedures for modelling realistic geometries. Specifically, the effects of tibial size and the distance of the fibular head location from the joint line were examined.

Geometries were derived from WBCT scans (OnSite, Carestream Health) of nine patients chosen by a Consultant Physiatrist and a Consultant Radiologist. The required ethical approval for the use of clinical data was received (reference: STUDY00146751). One knee scan was selected per patient and the patients were selected to represent a typical MOWHTO patient, following clinical guidelines for age, body mass index, alignment and OA grade [9]. The medial‐lateral width (MLW) at the tibial plateau had a mean value of 75 mm and a range from 67 to 84 mm. The fibular head tip was positioned anywhere between 3 and 12 mm below the tibial plateau.

**Figure 2:**
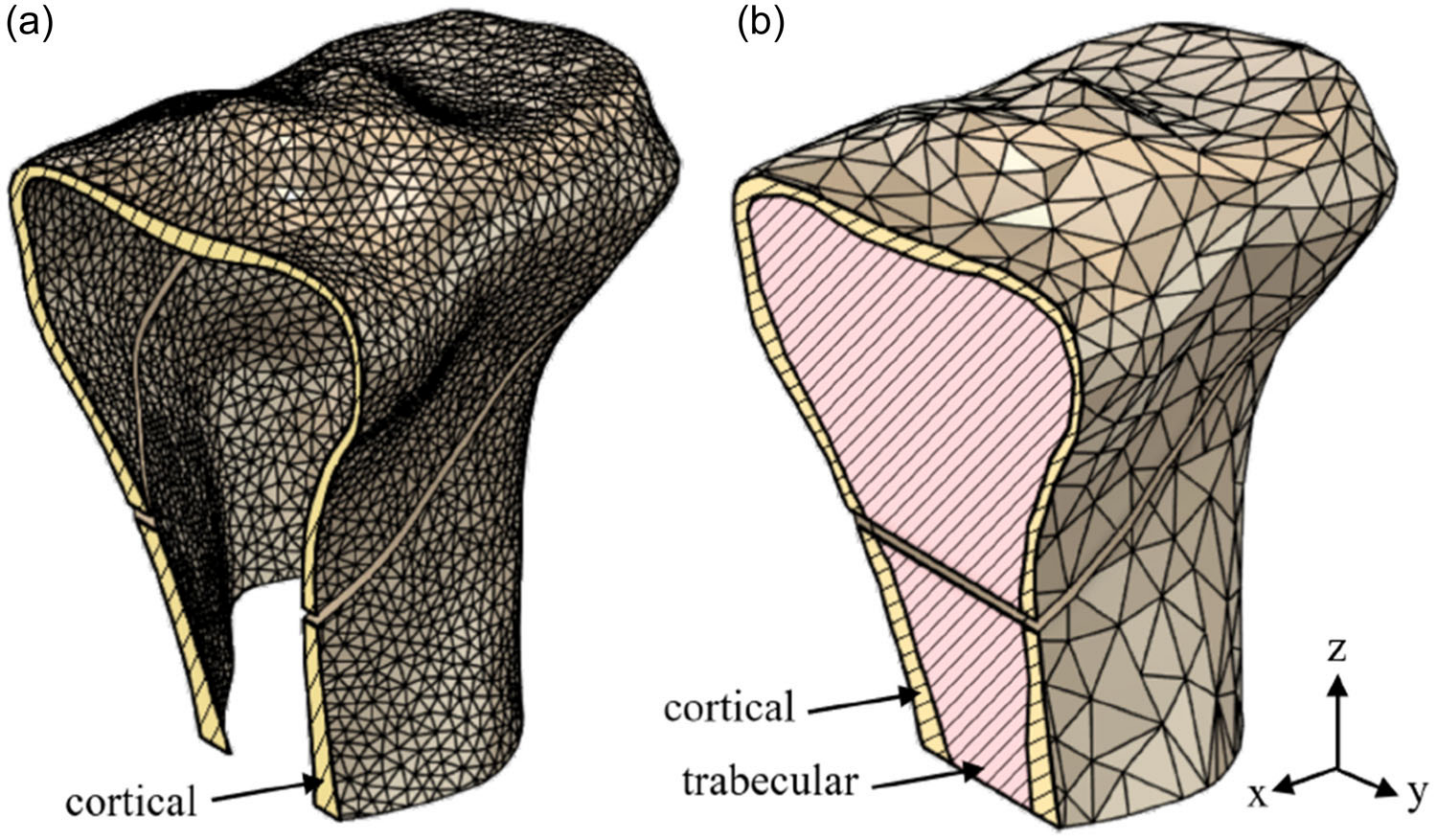
Models used for intra‐operative finite element analysis: (a) model for Part 1 consisting of the cortical bone, (b) model for Part 2 including cancellous as well as cortical bone.

**Figure 3:**
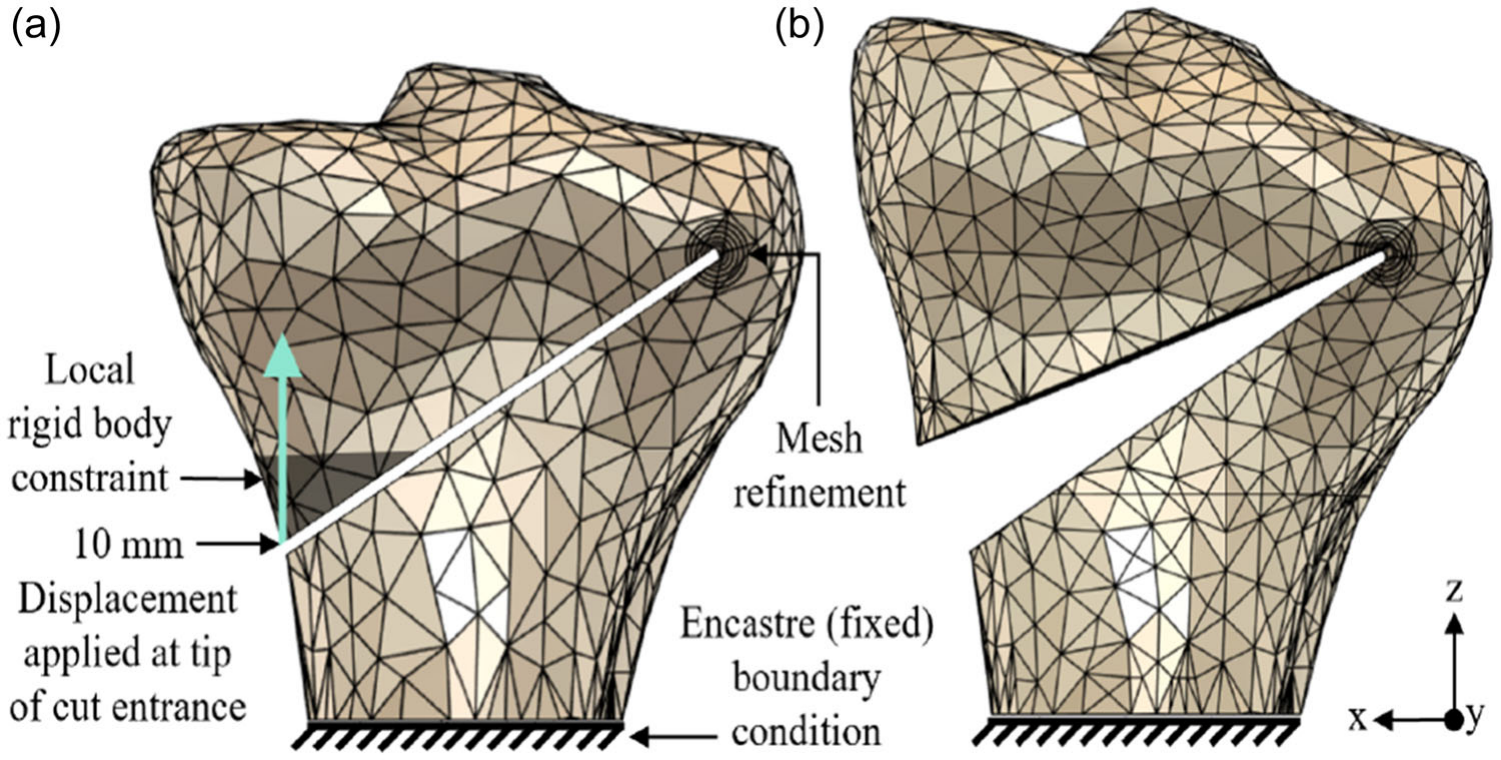
Constraints and loading used for the intra‐operative simulation for both parts of the finite element analysis study. The distal tibia was fixed at a location far enough from the lateral hinge and osteotomy cut so as not to affect the details at the apex. A 10 mm opening, representing in this case a 10‐degree correction, was created by applying a relative displacement on the cut face: (a) initial and (b) loaded configurations.

For each knee, a 3D model of the proximal tibia was developed, including only the cortical bone, to model the intra‐operative displacement at the osteotomy site (Figure 2a). Only the cortical bone was modelled in this part of the study to simplify the development of the FE models, essential with the various different geometries considered. This simplification brings with it the limitation of a loss of accuracy by excluding the effect of the cancellous bone. The cortical bone structure was modelled with a relatively fine mesh, as illustrated in Figure 2a. The mesh was further refined at the cut apex, with 20 elements through the thickness of the cortical shell. Material properties were assigned using Bonemat software (Istituto Ortopedico Rizzoli in Bologna, Italy) based on the CT image attenuation values. This software uses a nearly linear relationship between attenuation and bone density and an empirical relationship between density and elastic modulus, as described in detail in Schileo et al. [24]. Corresponding elastic modulus values were in the range of 10–17 GPa. Figure 3 illustrates the loading arrangement used for this part and for Part 2 of the FEA study. A 10 mm displacement was applied at the wedge opening, which in this case led to a typical osteotomy correction angle of 10 degrees.

In the first set of calculations, the osteotomy apex was located at a fixed point 10 mm from the lateral cortex and 17 mm distal to the joint line, as measured on the coronal/frontal plane. The resulting peak strain was correlated with the measured geometrical variations. Next, the location of the apex was scaled to bone size to examine whether scaling of the apex location in this way changed the resulting strains. The ratio of the hinge width to medial‐lateral width (*w*/MLW) was taken as 0.13 and the ratio of height (tibial plateau to apex) to medial‐lateral width (*h*/MLW) was 0.23, which corresponded to *w* = 10 mm and *h* = 17 mm on the average‐sized tibia.

Further variation was introduced to assess the impact of the apex cut geometry, by comparing cuts aiming either for the fibular head or towards the fibular circumferential line.

### Statistical analysis

Results were analysed using multiple linear regression and a single‐sided *t*‐test using Matlab software (Mathworks). The confidence level for statistical significance was set at 0.05. For the multiple regression analysis, the data was fitted with an equation of the form:

$$\text{strain}(\%)=\beta_{0}+\beta_{1}\times \text{tibia}\;\text{size}+\beta_{2}\times \text{fibular}\;\text{head}\;\text{location},$$

where the tibia size is defined as the maximum medial‐lateral width and the fibular head location is the distance of the fibular head tip from the tibial plateau. The analysis software calculates the *β* coefficients, the correlation coefficient *R* ^2^ for the goodness‐of‐fit of the model and *p*‐values quantifying the significance of each parameter in the fit.

### FEA: Part 2

Part 2 of the FEA study was used to enhance the accuracy of the modelling to further examine the effect of apex location on strains and to provide predictions of the fracture type. This model was developed from one of the CT scans used in Part 1 of the FEA. The model was again 3D but was more geometrically complex than that for Part 1, including both cortical and cancellous bone (Figure 2b). The cortical bone structure was modelled by a graded mesh, with the mesh seeding size varying from 5 mm away from the apex to 0.05 mm at the apex surface, as illustrated in Figure 2b. Material properties were based on those used by Kang et al. [10].

This model was first used to simulate an intra‐operative scenario, whereby a 10 mm opening was created, which in this case led to an approximately 10‐degree correction. The loading arrangement was the same as that used in Part 1 of the FEA study, see Figure 3. Width and height at the apex were varied to see the effect on predicted fracture type. Widths at the apex were varied from 6 to 16 mm, while heights were varied from 7 to 12 mm.

The same model was then used to conduct a postoperative analysis. Kang et al. [10] suggested an acceptable micromotion of 100–200 μm to ensure adequate stability and micromotion to allow the osteotomy site to heal. A load of 2500 N was applied to a proximal tibial model, which represented the maximum force during gait for a patient with a mass of 80 kg. The vertical loading direction was chosen for simplicity to give an indication of the relevant postoperative forces. With the component along the bone‐long axis varying with the cosine of the angle, this reasonably approximates the relevant loading. A TomoFix plate (DePuy Synthes) was included in the model so as to include the constraint on micromotion due to this component postoperatively. Micromotion, in micrometres (μm), was measured at the cut entry for models with hinge widths varying from 10 to 18 mm and for apex heights varying from 8 to 12 mm.

## RESULTS

### Proof of concept

Tables 1 and 2 present the observed Takeuchi failure type as a function of the apex location, for the Sawbones and Perspex specimens, respectively.

**Table 1:** The effect of the lateral hinge width on the Takeuchi failure type for the Sawbones specimens.

Lateral hinge width (mm)	4	7	10	13	16	19	22	25
Takeuchi failure type	I	I	I	I	I	III	III	III

*Note*: The distance from the joint line is 16 mm.

**Table 2:** The effect of the lateral hinge width and distance from the joint line on the Takeuchi failure type (I or III) for the Perspex specimens.

Lateral hinge width (mm)		4	7	10	13	16	19	22	25
Distance from joint line (mm)	13	I	I	I	III	III	III	III	III
	16	I	I	I	I	I	III	I	III
	19	I	I	I	I	I	I	I	I

The results for the Sawbones (Table 1) showed a switch from Takeuchi type I to type III failure as the hinge width increased above 16 mm. A similar switch from type I to type III failure was observed with the Perspex specimens when a critical value of the hinge width was exceeded, for the cases where the apex was either 13 or 16 mm from the joint line. For the case with the apex 19 mm from the joint line, all failures were type I. These results confirmed how clinically undesirable type III failure can be avoided by an appropriate choice of apex location.

**Figure 4:**
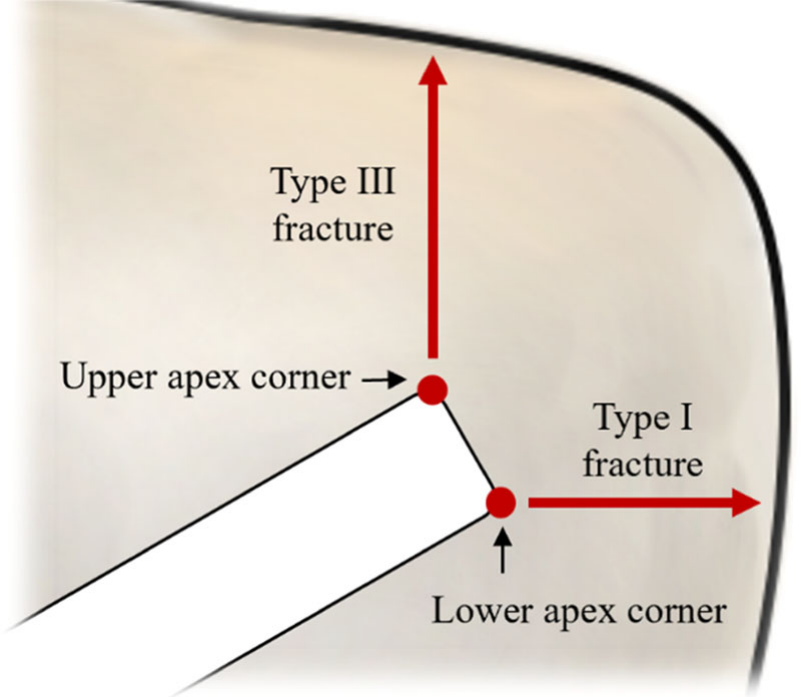
Schematic representation of findings from the proof‐of‐concept experimental study. For both Sawbones and Perspex specimens, every Takeuchi type I fracture was associated with the crack nucleating at the lower apex corner, while every Takeuchi type III was associated with the fracture nucleating at the upper apex corner.

Examination of the failed specimens from both the sawbones and Perspex experiments demonstrated that every Takeuchi type I fracture was associated with the crack nucleating at the lower corner of the rectangular apex, while every Takeuchi type III was associated with the fracture nucleating at the upper corner. This is shown schematically in Figure 4. This information was used to relate the stress patterns found in the FEA modelling to a predicted fracture type.

### FEA: Part 1

Models from nine patients were used to assess the variability of the maximum strain at the apex in an intraoperative analysis. A multiple linear regression was used to correlate the maximum strain for the nine tibia geometries with the tibia size and the fibular head location, for the case with a fixed apex location (*w* = 10 mm, *h* = 17 mm). Results are presented in the upper part of Table 3. The results showed a statistically significant negative correlation of maximum strain at the apex with both fibular head location and tibia size (defined by the maximum medial‐lateral width) (both *p* < 0.05). In other words, larger tibias with lower fibular heads were found to exhibit the lowest strains. The regression model explained 74% of the variation in the data, with the *R* ^2^ correlation coefficient equalling 0.74.

**Table 3:** Results of multiple linear regressions to fit the maximum strain at the apex for the nine tibias analysed in Part 1 of the finite element analysis.

Apex location	Variable	Coefficient (% strain/mm)	*p*‐Value	*R* ^2^ correlation coefficient
Fixed apex: *w* = 10 mm, *h* = 17 mm	Tibia size	$\beta_{1}=-0.73$	0.033	0.74
	Fibular head location	$\beta_{2}=-1.57$	0.017	
With scaling: *w* = 13%, *h* = 23% of the medial‐lateral width	Tibia size	$\beta_{1}=-0.03$	0.88	0.73
	Fibular head location	$\beta_{2}=-1.35$	0.011	

The corresponding multiple linear regression was also undertaken when the apex location was instead scaled with the maximum medial‐lateral width, taking *w*/MLW = 0.13 and ℎ/MLW = 0.23. Results are presented in the lower part of Table 3. Now the effect of fibular head location on strain was still found to be significant (*p* < 0.05), but the effect of tibia size on strain was not significant (*p*‐value equals 0.88). In other words, scaling the apex location with the medial‐lateral width ‘factors out’ the effect of tibia size. The correlation coefficient was very close to that for the fixed apex results, with *R* ^2^ for the overall model now equalling 0.73.

To assess the effect of tibia geometry on optimal cut location, results were compared when the osteotomy cut was directed either towards the fibular head tip or towards the circumferential line. The values of the peak strains averaged over the nine models were 17.8% and 20.6%, for the fibular head tip and circumferential line cut directions, respectively. A *t*‐test showed that this reduction in strain, given by aiming the cut at the fibular head tip, was statistically significant (*p* < 0.05).

### FEA: Part 2

**Figure 5:**
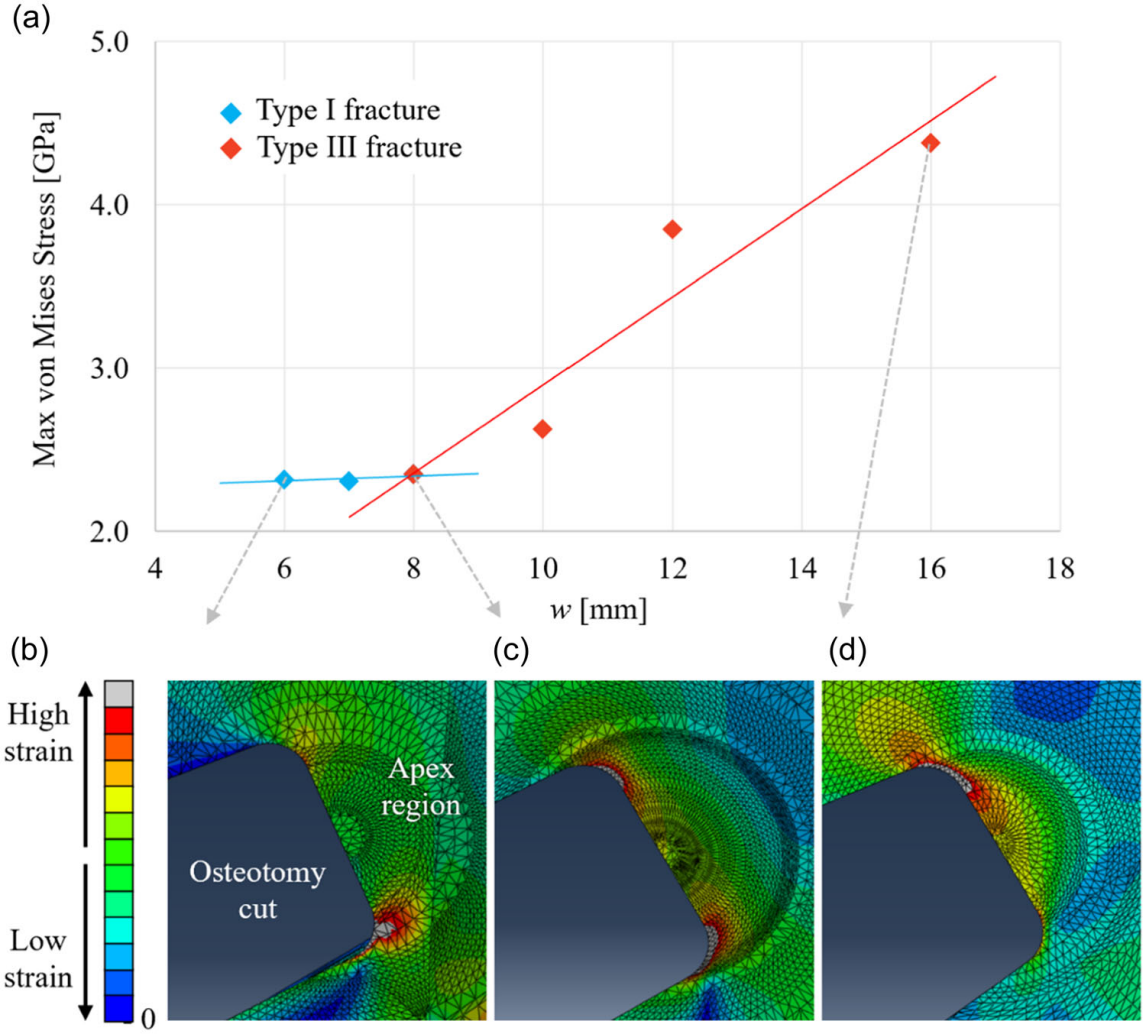
Prediction of failure from Part 2 of the finite element analysis study using the ‘strain at the apex’ classification method. (a) Variation of maximum stresses at the apex (stress in GPa on the *y‐*axis) with lateral hinge width (in mm on the *x‐*axis) for hinge height *h* = 8 mm, demonstrating the increased likelihood of a type III fracture with a wider hinge width. (b) Apex strains showing type I fracture for *w* = 6 mm. (c) Apex strains showing type I or type III fracture for *w* = 8 mm. (d) Apex strains showing type III fracture for *w* = 16 mm.

**Figure 6:**
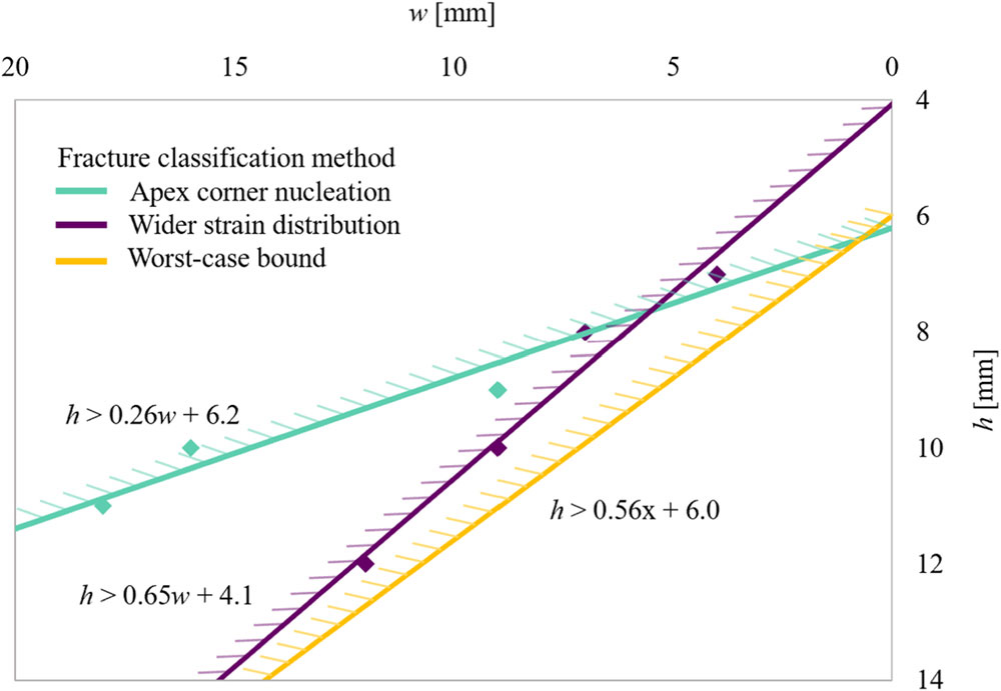
Graph (hinge width *w* in mm on the *x‐*axis; hinge height *h* from the tibial plateau in mm on the *y‐*axis) demonstrating the predicted type I to III transition bounds for the two different fracture classification methods. The region where a type III fracture is avoided lies to the bottom right of the figure, with a smaller lateral hinge width (smaller *w*) and with the apex further from the tibial plateau (larger *h*). The bottom line shows a conservative bound capturing the worst case for both fracture classification methods. Note that the *x*‐axis has been reversed so that the relative locations on this graph correspond to the physical locations on the tibia illustrations in this study.

Part 2 of the FE study used a more complex 3D model, incorporating cortical and cancellous bone. A prediction of failure type was made by examining the location of the maximum strain at the apex. This was motivated by the findings of the experimental proof‐of‐concept study, where it was observed that the failure type correlated with the fracture initiation site at the apex. To simplify the presentation of these results, absolute values (in terms of mm) for the location of the apex are presented in the first instance. Then, using the findings in the first section that the apex location should be scaled by the medial‐lateral width, the key findings are presented in this relative format. Figure 5 illustrates the ‘strain classification at the apex’ method to predict the fracture type, showing a switch in location of the maximum strain from the bottom to the top of the apex, corresponding to a predicted switch from type I to type III fracture. The corresponding results for the change in maximum von Mises stress with width are included in Figure 5. Based on the calculations of Part 2 of the FE study, with widths at the apex varied from 6 to 16 mm and heights varied from 7 to 12 mm, the limit for the optimal apex locations to prevent type III fracture could be calculated using the formula, ℎ > 0.26*w* + 6.2 (ℎ = height in mm; *w* = width in mm). To take into account possible crack blunting associated with viscoelastic or plastic deformation at the crack initiation site, an alternative failure characterisation criterion was considered by looking at the wider distribution of strains around the apex. With this ‘wider strain’ classification method, the optimal apex locations to prevent type III fracture corresponded to the relationship ℎ > 0.65*w* + 4.1. When these formulae are shown on a graph, they demonstrate the relationship between the two techniques for predicting type III fractures as width and height varies (Figure 6). The results from both criteria showed that a wider lateral hinge width and apex closer to the tibial plateau increased the likelihood of a type III fracture. Given the uncertainties in failure prediction, a more conservative bound of ℎ > 0.56*w* + 6.0 is recommended to avoid the clinically undesirable type III failure, which encompasses in a conservative manner both the bounds suggested. This is included as the lower bound, as shown in Figure 6.

Based on the finding of Part 1 of the FEA that the apex location should be scaled by the medial‐lateral width (i.e., working with *h* and *w* as a fraction of the medial‐lateral width MLW, which in this case was 72.5 mm), the corresponding scaled bound to avoid type III fracture is given as ℎ/MLW > 0.56*w*/MLW + 0.083.

**Figure 7:**
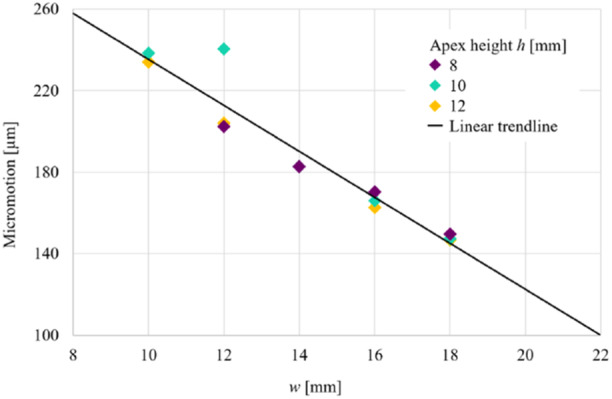
Graph demonstrating the results for micromotion (μm, *y‐*axis) versus hinge width *w* (mm, *x‐*axis). As hinge width increased, micromotion decreased. Values of the width *w* between 13 and 22 mm were found to keep micromotion between 100 and 200 μm as recommended by Kang et al. [10].

Results for the postoperative model confirmed that narrow hinges cause more micromotion. Micromotion decreased with increasing hinge width and micromotion remained relatively constant across different hinge heights. Specifically, choosing *w* between 13 and 22 mm was found to keep micromotion in the target range between 100 and 200 μm (Figure 7).

## DISCUSSION

This work was conducted to identify an optimum osteotomy apex location using FEA, allowing for adequate stability while reducing the risk of intra‐articular fractures occurring. Proof of concept experiments showed how changing the apex position resulted in a switch from type I to type III fracture. An FEA study was used to predict the effect of apex location on failure type for a range of apex locations and hence determine a bound on the apex location to avoid type III fracture. Postoperative loading was modelled to ensure that the proposed apex location gave appropriate micromotion for effective healing. The results support the hypothesis that bioengineering models using FEA can be used to predict the stresses at the osteotomy apex and hence develop guidelines for the location of the osteotomy apex so as to avoid the risk of clinically significant type III fractures during MOWHTO surgery while ensuring sufficient micromotion for postoperative healing.

Our study has investigated and identified an optimal location for the apex, based upon fracture risk prediction and micromotion at the osteotomy apex. We believe this finding is clinically useful due to the potential use of the apex point on preoperative 2D radiographs when planning surgery. Furthermore, we have shown that aiming for the fibular head is associated with less strain.

Both the sawbones and Perspex experiments demonstrated: (1) the wedge opening angle at failure decreased as the hinge width increased; (2) the failure load increased as the width of the lateral hinge increased. Perspex experiments additionally demonstrated: (3) a smaller wedge opening at the failure angle was required as the distance of the apex from the joint line increased; and (4) no clear influence on the failure load of the distance of the apex from the joint line.

A study conducted by Seo et al. [26] identified lateral cortex fracture (15.6%) as the most common complication in a cohort of 167 patients who underwent a MOWHTO. However, the incidence does vary within the literature between 3% and 30%, although these are based on radiographs [7], while Kim et al. [12] estimated that these fractures are nearly 10% higher when assessed with CT scans. Clinically, it is the experience of the senior surgical author that type II fractures occur most frequently as a postoperative phenomenon. This study relates to reducing intraoperative fracture risk and neither the experiments nor the FEA showed type II fracture with gap opening. Hence the key focus for determining an optimal apex location was avoiding type III fracture by identifying the transition from type I to type III fracture.

Kang et al. [10] suggested that the stability of the osteotomy postoperatively can be quantified by values of micromotion, the relative displacement between the upper and lower cut surfaces during postoperative loading. Similar to fracture healing [6], excessive micromotion and thus strain will lead to lower stability and greater fracture risk, whereas too little micromotion may slow down bone healing or lead to nonunion at the osteotomy site. Furthermore, Peez et al. [22] demonstrated that, independent from the osteotomy type, hinge fractures increase rotational displacement and reduce stiffness of the bone−implant construct by at least 70% in each rotational direction, with an additional plate leading to improved torsional stability. In our study, we included an assessment of stability following the suggestion of Kang et al. [10], albeit applying a more relaxed adherence to the proposed criteria.

We have mapped the transition of type I to type III fractures, based on the width and height of the apex location. The prediction of the type of fracture was based on the location of the maximum stress at the osteotomy apex, guided by the findings from the experimental part of the study. An overall bound limit of ℎ/MLW > 0.56 *w*/MLW + 0.083 has been suggested to avoid type III fracture (*h* = height and *w* = width expressed as a proportion of the MLW). Our postoperative FE results showed that the width of the lateral hinge has the greatest effect on postoperative stability, with wider hinges providing better stability.

The optimal points proposed in this analysis and recommended from the literature are similar in hinge height and differ more in hinge width. A study by Ogawa et al. [20] examined 82 osteotomies of which 11 resulted in fractures. They concluded that ‘a sufficient osteotomy involving both the anterior and posterior cortices, whose endpoint is at the level of the fibular head, should be performed when undertaking a MOWHTO if a lateral hinge fracture is to be avoided as a complication’. The study performed by Nakamura et al. [17] examined 111 patients, with 22 patients sustaining fractures, concluding that the relative risk of unstable fractures was significantly lower in the zone which was just above and lateral to the medial margin of the proximal tibiofibular joint. A more recent FEA study by Kyung et al. [14] assessed von Mises stresses at the lateral tibial cortex and concluded that the hinge should be located at the ‘upper end of the articular cartilage of the proximal tibiofibular joint’ to reduce fracture risk as this is ‘anatomically independent’ from the fibula. However, only three patient CT models and one control were analysed, and the osteotomies were planned as biplanar. Other more recent laboratory studies assessing hinge width include Saghaei et al. [23] who conducted a monoplanar MOWHTO study using 20 ostrich bones, varying the width from 9 to 32 mm and assessing for fracture presence and type at 10 mm of opening. They concluded that an increased lateral cortical hinge width was significantly associated with type II and III fractures, with these occurring at a mean width of 16 and 25 mm, respectively. In a synthetic model study, Turkmen et al. [29] assessed the maximum wedge gap that could be created at different hinge widths; they concluded that ‘higher angle corrections’ with less risk of fractures were achieved ‘by bringing the end point of the osteotomy closer to the lateral cortex’.

Our suggested location was shown not to be affected by the size of the tibia, and thus, scaling the suggested location with the medial‐lateral width allows generalisability to all tibias. In our provisional work, larger tibias with lower fibular heads were found to exhibit the lowest strains, but when the apex location was subsequently scaled to the medial‐lateral width (*w*/*MLW* = 0.13, ℎ/*MLW* = 0.23) there was no effect seen of tibia size on strain. Aiming for the fibular head tip was also associated with less strain compared to the circumferential line.

**Figure 8:**
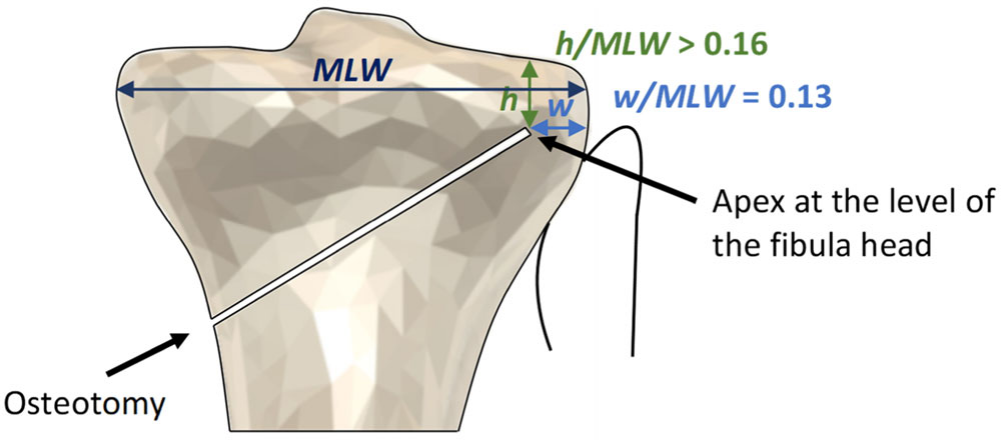
The optimal apex position (OAP) is determined by this finite element analysis study. This region can be used as a template upon preoperative imaging to plan the osteotomy apex location with the ratio *w/*MLW of the hinge width to the medial‐lateral width equal to 0.13 (blue line), while aiming in the first instance for the fibula head. The ratio *h/*MLW of the height of the apex from the tibial plateau to the medial‐lateral width should not fall below 0.16 (green line), aiming below the fibula head if necessary to meet this requirement.

Thus, we believe our results can be used to identify a simple clinical guideline for the OAP as an evidence base for MOWHTO surgery. The suggestion is that the apex should be at the height of the fibular head and with the ratio of the apex width to medial‐lateral width *w/*MLW = 0.13. For the calculations of Part 2 of the FEA, this recommendation ensures that type III fracture is avoided as long as the ratio ℎ/MLW of the distance of the fibular head from the tibial plateau to the medial‐lateral width exceeds 0.16 (using the bound ℎ/MLW > 0.56 *w*/MLW + 0.083). For a typical medial‐lateral width of 72.5 mm, this corresponds to the fibular head being more than 12 mm from the tibial plateau, which would generally be the case. If taking the apex at the fibular head gives ℎ/MLW < 0.16, then the apex location should be moved down so that ℎ/MLW = 0.16. This guideline priorities a simple‐to‐apply rule which avoids the critical type III fracture and reduces apex tip stresses by minimising *w*, at the expense of somewhat exceeding the suggested micromotion range. Indeed, Yoshida et al. [32] conducted a retrospective cohort study and concluded that, ‘the hinge position should be placed at a certain distance from the articular surface to avoid type III LHF, especially in participants with higher fibular head position, even if the hinge position is located in the safe zone.’ Our findings have suggested ‘a certain distance’, which is applicable to all patients. In fact, the predicted micromotion taken from the calculations of Part 2 of the FEA equals 235 μm for *w/*MLW = 0.13, exceeding by 18% the recommended microstrain. However, given the uncertainties in these calculations and the priority of avoiding type III fracture during surgery, we believe that this is an acceptable outcome. These findings for the optical apex location can be used as a template for preoperative imaging to plan the osteotomy apex as illustrated in Figure 8.

We recognise that optimising the apex position is not the only solution to reducing fracture risk. Other FEA work analysing techniques to reduce fractures has also been conducted. The use of an apex drill hole by Bostrom et al. [2] demonstrated that a 4 mm apical drill hole centred 10 mm from the lateral cortex reduced bone strain and led to improved control. Alternative modifications include drilling a pilot hole through the apex of the cut and adapting the use of a hinge wire or screw. Kaze et al. [11] and Carranza et al. [3] have undertaken FEA and experimental studies to investigate the effect and found a very minor improvement [11] or an increased fracture risk [3], respectively.

Didier et al. [5] investigated whether varying the speed at which the osteotomy site is opened correlates with fracture risk and did not find an association, while Soykan et al. [27] identified ‘a significant reduction in the force required for distraction’ in cases associated with a lateral hinge fracture, thus suggesting the potential need to measure forces when distracting the osteotomy site to act as a warning to surgeons. Koh et al. [13] conducted a cohort study, showing the use of a k‐wire reduced the incidence of lateral hinge fractures in small (<10 mm) and large (>10 mm) corrections. Finally, computer‐assisted MOWHTO surgery has not shown superior clinical outcomes, while no specific analysis has been conducted in the literature on lateral hinge fracture risk and the use of navigation [7, 31].

Limitations of the model include the number of patient geometries used in the data and the simplifications made in defining the osteotomy location. Systematic changes in cortical thickness, for example, with age, as well as normal variability between people, could affect the strain predictions. Although the von Mises stress measure is appropriate for the failure of ductile materials such as metals, we have used this as a simple way of characterising the local loading in the bone associated with deformation ahead of a crack tip. It is possible that other measures of local loading, for example, the maximum tensile stress component, could be more representative, but the location of the maxima for these two components is likely to be closely correlated so that the simplification is appropriate. More extensive modelling in the future could consider a fracture model at the apex or include the time dependence of loading rate and viscoelastic bone properties. This could be used to define the critical conditions for either type I or type III fracture, rather than simply developing a criterion to avoid type III fracture. Further modelling could also include analysis of hinge wires/screws. Finally, we recognise that the apex in this study has predominantly been investigated and defined in the coronal plane as a 2D structure, while in vivo, its location also has a sagittal element. However, our study has replicated what takes place during preoperative planning when the location of an apex is identified in the coronal plane on a plain radiograph, thus making these findings clinically translatable.

## CONCLUSIONS

To reduce the risk of type III fractures when performing MOWHTO, the OAP is located such that the ratio *w*/MLW of the hinge width to the medial‐lateral tibia width equals 0.13. The apex of the cut should be at the fibular head, with the proviso that the ratio ℎ/MLW of the height of the apex from the tibial plateau to the medial‐lateral width should not be less than 0.16. In this case, the apex should be moved down such that ℎ/MLW = 0.16. These recommendations can be used irrespective of the size of the tibia.

## AUTHOR CONTRIBUTIONS

Humza T. Osmani contributed to the interpretation of data and drafted the manuscript. Radhika Gupta, Rosemary Earl and Stanisław Tomaszczyk carried out experiments, analysis, interpretation of data and contributed to drafting the manuscript. Tom Turmezei advised on and reviewed image data, contributed to the interpretation of data and reviewed the manuscript. Neil A. Segal provided imaging for the experiments, advised on and reviewed image data, contributed to the interpretation of data and reviewed the manuscript. Michael Sutcliffe participated in the design of the study, coordination, experiments, data analysis, interpretation and co‐drafted the manuscript. Joel Melton participated in the design of the study, coordination, data analysis, interpretation and co‐drafted the manuscript.

## CONFLICT OF INTEREST STATEMENT

The authors declare no conflict of interest.

## ETHICS STATEMENT

Received from the University of Kansas Medical Center (reference: STUDY00146751).

## Data Availability

The datasets used and/or analysed during the current study are available from the corresponding author upon reasonable request.
